# Do Exophytic and Endophytic Patterns in Borderline Ovarian Tumors Have Different Prognostic Implications? A Large Multicentric Experience

**DOI:** 10.3390/jcm12103544

**Published:** 2023-05-18

**Authors:** Vito Andrea Capozzi, Elisa Scarpelli, Luciano Monfardini, Vincenzo Dario Mandato, Carla Merisio, Stefano Uccella, Giulio Sozzi, Marcello Ceccaroni, Vito Chiantera, Giovanna Giordano, Luigi Della Corte, Carmine Conte, Stefano Cianci, Tullio Ghi, Roberto Berretta

**Affiliations:** 1 Department of Medicine and Surgery, University Hospital of Parma, 43125 Parma, Italy; 2 Unit of Obstetrics and Gynecology, Azienda USL-IRCCS di Reggio Emilia, 42122 Reggio Emilia, Italy; 3 Department of Obstetrics and Gynecology, AOUI Verona, University of Verona, 37126 Verona, Italy; 4 Department of Gynecologic Oncology, University of Palermo, 90127 Palermo, Italy; 5 Department of Obstetrics and Gynaecology, Gynaecologic Oncology, and Minimally Invasive Pelvic Surgery, International School of Surgical Anatomy, Sacred Heart Hospital, 37024 Negrar, Italy; 6 Pathology Unit, Department of Medicine and Surgery, University of Parma, 43125 Parma, Italy; 7 Department of Neuroscience, Reproductive Sciences and Dentistry, School of Medicine, University of Naples Federico II, 80131 Naples, Italy; 8 Department of General Surgery and Medical-Surgical Specialties, Institute of Obstetrics and Gynecology, A.O.U. Policlinico Rodolico, San Marco, University of Catania, 95125 Catania, Italy; 9 Unit of Gynecology and Obstetrics, Department of Human Pathology of Adult and Childhood “G. Barresi”, University of Messina, 98121 Messina, Italy

**Keywords:** borderline ovarian tumor, exophytic pattern, endophytic pattern, tumor growth, BOT

## Abstract

Borderline ovarian tumor (BOT) accounts for 15–20% of all epithelial ovarian tumors. Concerns have arisen about the clinical and prognostic implications of BOT with exophytic growth patterns. We retrospectively reviewed all cases of BOT patients surgically treated from 2015 to 2020. Patients were divided into an endophytic pattern (with intracystic tumor growth and intact ovarian capsule) and an exophytic pattern (with tumor growth outside the ovarian capsule) group. Among the 254 patients recruited, 229 met the inclusion criteria, and of these, 169 (73.8%) belonged to the endophytic group. The endophytic group showed more commonly an early FIGO stage than the exophytic group (100.0% vs. 66.7%, *p* < 0.001). Furthermore, tumor cells in peritoneal washing (20.0% vs. 0.6%, *p* < 0.001), elevated Ca125 levels (51.7% vs. 31.4%, *p* = 0.003), peritoneal implants (0 vs. 18.3%, *p* < 0.001), and invasive peritoneal implants (0 vs. 5%, *p* = 0.003) were more frequently observed in the exophytic group. The survival analysis showed 15 (6.6%) total recurrences, 9 (5.3%) in the endophytic and 6 (10.0%) patients in the exophytic group (*p* = 0.213). At multivariable analysis, age (*p* = 0.001), FIGO stage (*p* = 0.002), fertility-sparing surgery (*p* = 0.001), invasive implants (*p* = 0.042), and tumor spillage (*p* = 0.031) appeared significantly associated with recurrence. Endophytic and exophytic patterns in borderline ovarian tumors show superimposable recurrence rates and disease-free survival.

## 1. Introduction

Borderline ovarian tumor (BOT) accounts for 15–20% of all epithelial ovarian neoplasms [1,2,3]. Differently from malignant epithelial ovarian tumors, BOTs are characterized by atypical epithelial proliferation in the absence of stromal invasion [4]. BOTs usually affect younger women compared to invasive carcinoma; in particular, one-third of patients diagnosed with BOT are younger than 40 years [5,6]. Moreover, the five-year overall survival reaches 95–97% [7,8,9], and in most cases, BOTs are diagnosed at an early stage [10,11].

**Figure 1:**
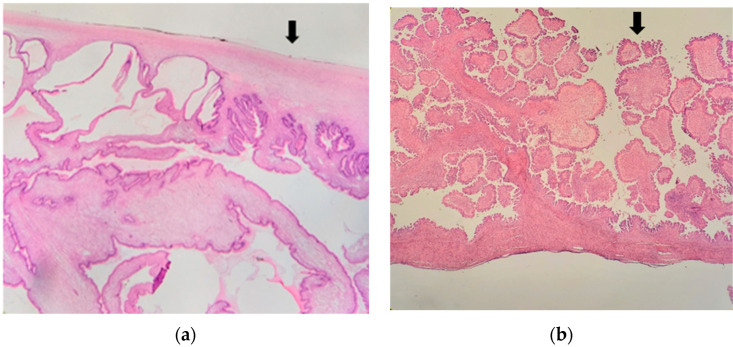
Microscopic endophytic pattern (**a**) and Exophytic pattern (**b**). The flash indicates, respectively, the regular (**a**) and irregular (**b**) aspects of the ovarian surface.

The most frequent histologic subtypes are serious (sBOT) and mucinous (mBOT) tumors, which represent 53.3% and 42.5% of cases, respectively [12,13]. sBOT can present as unilocular solid or multilocular solid lesions with multiple papillary projections within the cyst [14]. Frequently the tumor is “stromal proliferative” or “cyst forming”. In these latter cases, the tumor surface is regular, a cystic mass is present, and the pattern of growth is defined as endophytic (Figure 1a) [15]. Less frequently, sBOT shows a proliferative pattern and arises from the ovarian surface. In these cases, the tumor has papillary excrescences on the ovarian surface, while the ovary can be normal in size and shape [15,16]. In surface proliferative tumors, the neoplasm surface is irregular, and the pattern of growth is also called exophytic (Figure 1b).

As for sBOT, mBOTs present papillary intracystic infoldings with a smooth ovarian surface in most cases [17,18,19]. However, two types of mBOT have been described in the literature, i.e., the gastrointestinal and the endocervical one. The gastrointestinal subtype is the most frequent entity and appears as a unilateral large multilocular mass with a smooth ovarian surface. Conversely, the endocervical mBOT is less common and presents as a bilateral mass with an exophytic pattern of growth [20,21]. 

Concerns have arisen about the clinical and prognostic implications of BOT with exophytic growth patterns among clinicians. Intuitively, the exophytic pattern could be associated with a more advanced stage, greater peritoneal dissemination, and a greater relapse rate with a worse prognosis. However, to the best of our knowledge, no studies have investigated and compared the outcomes of the two BOT growth patterns.

This study aims to report the clinical characteristics and prognosis of BOT patients with exophytic and endophytic patterns who underwent surgical treatments.

## 2. Materials and Methods

We retrospectively reviewed all cases of histologically confirmed BOT patients treated at the University Hospital of Parma, the University Hospital of Verona, the IRCCS Sacred Heart Hospital Don Calabria of Negrar, Civico Hospital of Palermo, and Arcispedale Maria Nuova of Reggio Emilia, from 2015 to 2020.

All BOT patients who underwent fertility-sparing or non-fertility-sparing treatment were included in the analysis. Complete surgical staging included hysterectomy, bilateral salpingo-oophorectomy, omentectomy, peritoneal biopsies, and peritoneal washing, in accordance with international guidelines, in post-menopausal women [22]. The fertility-sparing procedure included the preservation of at least part of one ovary and the uterus, and it was performed in patients of reproductive age with no evidence of extra-ovarian disease. Patients with ovarian cancer at the final diagnosis, with missing pathological data, or <18 years old were excluded. Data were collected regarding patients’ characteristics, ultrasound aspects of the lesion, type of surgical treatment, histological subtype, stage at diagnosis, recurrences, and disease-free survival (DFS). Regardless of the histological type, patients were divided into an endophytic pattern (with intracystic tumor growth and intact ovarian capsule) and an exophytic pattern (with tumor growth outside the ovarian capsule). Microscopic evaluation of the ovarian surface was performed by dedicated pathologists in each participating center. Pathologic evaluation was carried out according to the latest available WHO Classification of Tumours of the Female Genital Organs [23,24]. For serous histology, peritoneal implants were classified as invasive or non-invasive. International Ovarian Tumor Analysis (IOTA) terminology was used to describe the sonographic characteristics of ovarian lesions [25]. The International Federation of Gynaecology and Obstetrics (FIGO) staging system 2014 was used to classify BOT tumors [26]. DFS was considered from the main surgery to the day of relapse in case of recurrence or to the last follow-up in case of no relapse. Ca125 was considered abnormal for levels > 35 U/mL. Gynecological examination, transvaginal ultrasound, and neoplastic markers were performed every three months in the first two years of follow-up, then every six months for three years.

The study was approved by the Parma Ethics Committee under code 343/2021/OSS/AOUPR. All patients who met the inclusion criteria had provided written consent for the use of their anonymized data for scientific purposes. 

For the statistical analysis, the Statistical Package for Social Science (SPSS) version 25 was used. Continuous variables were presented as the median. Categorical variables were presented as numbers and percentages. Chi-square or Fisher exact tests, when appropriate, were used to compare categorical variables. The Mann–Whitney test was used as a nonparametric equivalent test if needed. Kaplan Meier curves and Cox’s regression were used to analyze disease-free intervals. A logistic regression model was used for multivariable analysis. Differences were considered statistically significant with *p* < 0.05.

## 3. Results

Among the 254 patients initially recruited, 229 met the inclusion criteria, and of these, 169 (73.8%) belonged to the endophytic group. Patients’ characteristics are summarized in Table 1. Patients in the endophytic group were older (50 vs. 41 years, *p* = 0.001), less frequently nulliparous women (38.5% vs. 58.3%, Odds Ratio (OR) 0.659; 95% Confidence Interval (CI) 0.495–0.878, *p* = 0.008), more often with BMI > 30 Kg/m^2^ (17.8% vs. 5%, *p* = 0.0116, OR 3.5; 95% CI 1.125–11.209) and in post-menopause (88 vs. 16 patients, 52.1 vs. 26.7%; *p* 0.001, OR 1.953; 95% CI 1.253–3.004).

Table 2 shows patients’ pathological data. Serous histology was present in 134 cases (58.5%), mucinous histology in 87 patients (38.0%), and 8 patients had other BOT histology (3.5%). Serous histology was significantly more common in the exophytic vs. endophytic group (78.3% and 51.5%, respectively, *p* < 0.001, OR 3.4, 95% CI 1.719–6.755). Conversely, the mucinous histology was significantly associated with the endophytic pattern of growth (44.4% vs. 20.0%, *p* = 0.001, OR 2.2, 95% CI 1.302–3.783).

In our series, 136 patients underwent minimally invasive surgery without significant difference depending on the pattern of presentation (56.2% vs. 68.3%, *p* 0.101). Fertility-sparing surgery was performed in 30.2% of cases in the endophytic group and 48.3% of cases in the exophytic group (*p* = 0.011, OR 2.164, 95% CI 1.184–3.958). Detailed FIGO stages and patient distribution were summarized in Table 2. As concerns the surgical stage, the endophytic group showed more commonly an early FIGO stage than the exophytic group (100.0% vs. 66.7% of FIGO stage I, respectively, *p* < 0.001, OR 1.500, 95% CI 1.254–1.794). Furthermore, tumor cells in peritoneal washing (20.0% vs. 0.6%, *p* < 0.001, OR 42.000, 95% CI 5.326–331.203) and elevated Ca125 levels (51.7% vs. 31.4%, *p* = 0.003, OR 2.594, 95% CI 1.377–4.887) were more frequently observed in the exophytic group. Eleven patients (4.5%) with peritoneal implants were found in the entire series, all belonging to the exophytic group (*p* < 0.001, OR 1.224, 95% CI 1.086–1.380). Of these, three (1.3%) were invasive peritoneal implants (*p* = 0.003, OR 1.053, 95% CI 0.993–1.116). Intraoperative tumor spillage was reported in 8.9% of cases (*n* = 15) in the endophytic pattern group and 8.3% (*n* = 5) of cases in the exophytic pattern group (*p* = 0.121).

The sonographic characteristics of ovarian lesions are summarized in Table 3. No statistical differences in maximum tumor diameter (82 mm vs. 67.5 mm, *p* = 0.564), median diameter of the solid component (20 mm vs. 33 mm, *p* = 0.070), cyst vascularization (*p* = 0.404), presence of papillae (50.9% vs. 50.0%, *p* = 0.991), and >10 loculi expression (12.4% vs. 13.3%, *p* = 0.823) were found between the endophytic and exophytic groups, respectively. Nevertheless, patients with endophytic tumor growth more often had maximum lesion diameter > 100 mm (41.4% vs. 26.7%, *p* = 0.043, OR 1.553, 95% CI 0.984–2.451) and less frequently solid component > 10 mm (34.9% vs. 50.0%, *p* = 0.017, OR 2.093, 95% CI 1.133–3.866) than patients with exophytic tumor growth. 

**Figure 2:**
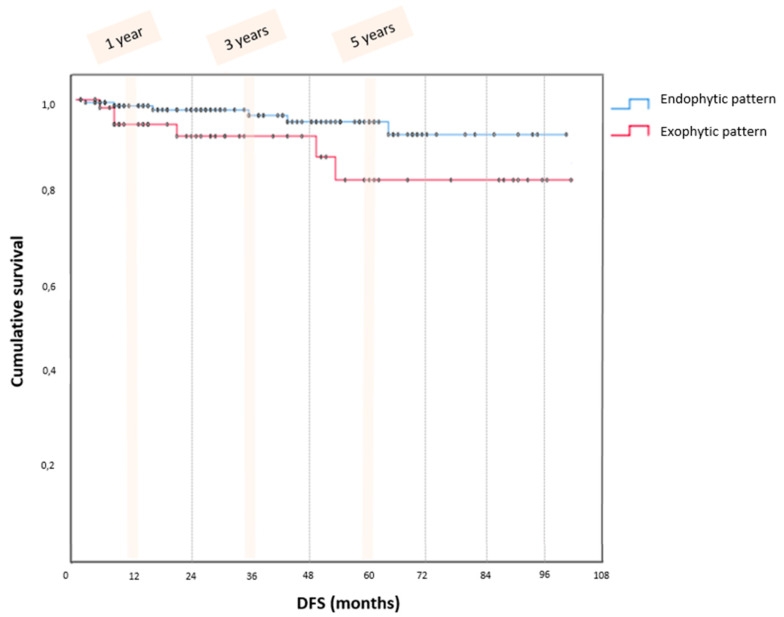
Kaplan Meier curves.

Finally, survival analysis showed 15 (6.6%) total recurrences, 9 (5.3%) in the endophytic and 6 (10.0%) patients in the exophytic group (*p* = 0.213) with a median follow-up of 45 months (6–120). Kaplan Meier curves (Figure 2) showed no significant differences (*p* = 0.076) in DFS between the endophytic and exophytic patterns at 1-year (99.4 vs. 94.5%), 3-year (98.6 vs. 91.9%), and 5-year (96.5 vs. 82.2%). 

At multivariable analysis woman’s age (*p* = 0.001), FIGO stage (*p* = 0.002), fertility-sparing surgery (*p* = 0.001), presence of invasive implants (*p* = 0.042), and tumor spillage (*p* = 0.031) but not the pattern of tumor growth appeared significantly associated with recurrence. 

## 4. Discussion

### 4.1. Summary of Main Results

The present study did not find a significant difference in the recurrence rate and DFS between the endophytic and exophytic BOT patterns. Nevertheless, the exophytic pattern showed a significant association with the advanced FIGO stage, peritoneal implants, the presence of neoplastic cells in peritoneal washing, and abnormal Ca125 levels. 

Contrary to our expectations, the exophytic pattern was not a worsening factor in patients’ survival. Although the exophytic variant correlated with more aggressive behavior than the endophytic pattern, the multivariable analysis excluded the tumor growth pattern as an independent variable influencing recurrence.

### 4.2. Results in the Context of Published Literature

Intuitively, tumor growth above the ovarian surface is expected to promote the spillage of the neoplastic cells into the abdominal cavity. Consequently, neoplastic cells could rest on the peritoneal surfaces leading to a local inflammatory response with Ca125 elevation, neoplastic cells within the peritoneal washing, and subsequent peritoneal neoplastic infiltration [27,28,29,30]. All these pathogenetic events would explain the significant association of exophytic BOT with negative prognostic factors. Despite this, the exophytic pattern did not change the patient’s prognosis. Two possible explanations could justify this result. First, the FIGO stage is the main prognostic factor in BOT patients [31,32]. Therefore, although the endophytic pattern is FIGO stage IA and the exophytic pattern is FIGO stage IC2, both patterns fall into FIGO stage I. Then, the type of surgical treatment does not change the patients’ survival but does influence the rate of recurrence [12,32]. In our series, the type of surgical treatment (fertility-sparing vs. non-fertility-sparing) was not changed with the BOT pattern found intraoperatively. Both these aspects could justify the overlapping prognosis between the two study groups. 

Finally, a statistical issue should be considered. BOT tumors are neoplasms with an excellent prognosis. Therefore, a low overall recurrence rate was reported in our series despite the large number of cases analyzed. Consequently, the rarity of the events, in the absence of differences in surgical approach and type of surgery, may justify the lack of significance in DFS between the two groups.

### 4.3. Strengths and Weaknesses

The present study reports a large, multicentre case series of patients with BOT who underwent adequate surgical treatment with long follow-ups. To the best of our knowledge, no author had previously reported a BOT classification based on tumor growth morphology. We acknowledge that the present study has limitations inherent in its retrospective nature, one of them being the lack of unambiguous evaluation of specimens by a single dedicated pathologist. Furthermore, the rarity of the recurrence event and the absence of differences in surgical approach and type of surgery between the two study groups were limitations of the study.

### 4.4. Implications for Practice and Future Research

Following our results, BOT clinical management and follow-up are not to be influenced by tumor pattern of growth. Conversely, postoperative follow-up and counseling should be based on the previously mentioned risk factors. However, due to the more common association of the exophytic pattern with the presence of peritoneal implants, a careful peritoneal evaluation should be performed if the exophytic pattern is found intraoperatively. 

In line with our results, Longacre et al. [33] reported that the exophytic pattern was associated with invasive and non-invasive peritoneal implants. Furthermore, the authors characterized exophytic BOTs as neoplasms with an intermediate prognosis between benign and malignant diseases. Moreover, such histologic entities posed problems in taxonomy, differential diagnosis, and prognosis.

The previous literature investigated factors correlating with the prognosis of BOT patients. In line with our results, peritoneal implants, age, FIGO stage, and conservative treatment were considered as independent factors worsening prognosis [34,35,36]. 

To date, two hypotheses on the pathogenesis of peritoneal implants have been formulated. The most supported hypothesis states that peritoneal implants would be due to neoplastic cells exfoliating from the tumor and resting on the peritoneum [29,30,37]. On the other, some authors suggest the ex novo origin of the implant from the peritoneum [38,39,40]. In any case, mortality rates of 15–34% and 4% have been reported for invasive and non-invasive implants, respectively [9,41]. Furthermore, in line with our results, Ozenne et al. reported the presence of peritoneal implants as an independent factor of recurrence (OR = 5.52, 95% CI 1.8–17.0, *p* = 0.003) [42].

Finally, recent evidence suggests that BOTs would have a silent attitude for several years, then a molecular trigger would increase cell replication with subsequent evolution into carcinoma or a tendency to recurrence [43]. In 2020, Genestie et al. identified p53 and KRAS mutation as useful identifying biomarkers of carcinoma and BOT, respectively [36]. In this context, molecular characterization of exophytic BOTs would be useful to better elucidate tumor pathogenesis and prognosis. Indeed, rapid molecular tests for clinical purposes have already been proposed [44,45]. 

## 5. Conclusions

Endophytic and exophytic patterns in borderline ovarian tumors show superimposable recurrence rates and DFS. The exophytic pattern was associated with advanced FIGO stage, peritoneal implants, the presence of neoplastic cells in peritoneal washing, and abnormal Ca125 levels. The intraoperative finding of exophytic BOT should not change patients’ clinical management, but a thorough evaluation of peritoneal surfaces is strongly suggested.

## Figures and Tables

**Table 1:** Patients characteristics.

	Total n; %	Endophytic Endophytic Pattern n; %	Exophytic Pattern n; %	*p*
	229; 100.0	169; 73.8	60; 26.2	
**Median Age**	49 (18–90)	50 (18–90)	41 (18–82)	**0.001**
**Median BMI**	25 (17–50)	25 (17–40)	24 (19–50)	0.680
**BMI > 30**	33; 14.4	20; 17.8	3; 5.0	**0.016**
**Menopausal state**	104; 45.4	88; 52.1	16; 26.7	**0.001**
**No Parity**	100; 43.7	65; 38.5	35; 58.3	**0.008**
**Abnormal Ca125**	84; 36.7	53; 31.4	31; 51.7	**0.003**
**Fertility sparing surgery**	80; 34.9	51; 30.2	29; 48.3	**0.011**

**Table 2:** Pathologic characteristics.

	Total n; %	Endophytic Pattern n; %	Exophytic Pattern n; %	*p*
**Histologic subtype**				
**Serous**	134; 58.5	87; 51.5	47; 79.7	**<0.001**
**Mucinous**	87; 38.0	75; 44.4	12; 20.3	**0.001**
**Others**	7; 3.1	7; 4.1	0; -	0.109
**FIGO stage**				**<0.001**
**IA**	160; 69.9	156; 92.3	4; 6.7	
**IB**	14; 6.1	13; 7.7	1; 1.7	
**IC**	35; 15.3	0; -	35; 58.3	
**IIA**	4; 1.7	0; -	4; 6.7	
**IIB**	1; 0.4	0; -	1; 1.7	
**IIIB**	4; 1.7	0; -	4; 6.7	
**IIIC**	11; 4.8	0; -	11; 18.3	
**Peritoneal implants**	11; 4.5	0;-	11;18.3	**<0.001**
**Invasive implants**	3; 1.3	0; -	3; 5.0	**0.003**
**Pcytologycitology in peritoneal washing**	13; 5.7	1; 0.6	12; 20.0	**<0.001**

**Table 3:** Sonographic features.

	Total n; %	Endophytic Pattern n; %	Exophytic Pattern n; %	*p*
**Median diameter**	80 (15–370)	82 (18–370)	67.5 (15–270)	0.564
**Diameter > 100 mm**	86; 37.6	70; 41.4	16; 26.7	**0.043**
**Diameter solid component mm**	22 (5–180)	20 (5–85)	33 (5–180)	0.070
**Solid component > 10 mm**	89; 40.1	59; 34.9	30; 50.0	**0.017**
**Color Score **				0.404
**Color score 1**	105; 45.9	81; 47.9	24; 40.0	0.606
**Color score 2**	90; 39.3	65; 38.5	25; 41.7	0.383
**Color score 3**	21; 9.2	16; 9.5	5; 8.3	0.847
**Color score 4**	3; 1.3	1; 0.6	2; 3.3	0.100
**N of locules**				
**<10 Loculi**	66; 28.8	52; 30.8	14; 23.3	0.301
**>10 Loculi**	29; 12.7	21; 12.4	8; 13.3	0.823
**N of papillae**				
**No papillae**	116; 50.7	86; 50.9	30; 50.0	0.991
**<4 papillae**	96; 41.9	72; 42.6	24; 40.0	0.792
**>4 papillae**	9; 3.9	6; 3.6	3; 5.0	0.603
**Ascitis**	19; 8.3	14; 8.3	5; 8.3	0.983

## Data Availability

Data available on request due to ethical restrictions.
